# A CT-Based 3D Anatomical Mapping Study of Right Posterior Portal and Hepatic Venous Patterns: Revisiting Liver Segmentation and Its Surgical Implication

**DOI:** 10.3390/jcm15156093

**Published:** 2026-08-05

**Authors:** Takashi Imanaka, Zenichi Morise, Hiroyuki Kato, Kenichi Nakamura, Tetsuya Koide, Kazuhiro Matsuo, Tomoyoshi Endo, Koji Morohara, Akihiko Horiguchi, Hidetoshi Katsuno

**Affiliations:** 1 Department of Surgery, Fujita Health University School of Medicine, Okazaki Medical Center, Okazaki 444-0827, Aichi, Japan; takashi.imanaka@fujita-hu.ac.jp (T.I.); naka-ken@fujita-hu.ac.jp (K.N.); tetsuya.koide@fujita-hu.ac.jp (T.K.); kzhrmto@fujita-hu.ac.jp (K.M.); tomtom23@fujita-hu.ac.jp (T.E.); koji.morohara@fujita-hu.ac.jp (K.M.); katsuno@fujita-hu.ac.jp (H.K.); 2 Department of Gastroenterological Surgery, Fujita Health University School of Medicine, Bantane Hospital, Nagoya 454-8509, Aichi, Japan; hiroyuki.kato@fujita-hu.ac.jp (H.K.); akihori@fujita-hu.ac.jp (A.H.)

**Keywords:** anatomical liver resection, hepatic vein, liver segments, portal vein, right posterior section, minimally invasive liver resection

## Abstract

**Background:** Classical Couinaud’s segmentation assumes the dichotomous division of the right posterior portal vein (RPPV) into P6 and P7. However, increasing imaging evidence suggests substantial anatomical variability, limiting the intraoperative identification of segmental borders and providing guidance for subsegmental resection. **Methods:** We retrospectively analyzed contrast-enhanced five-phase computed tomography (CT)-based three-dimensional reconstructions from 100 consecutive eligible cases (2020–2025). Third-order RPPV branching patterns were classified into three types: bifurcation (PV-type 1), loops (PV-type 2), and others (PV-type 3). We measured the 3D distances from the posterior portal origin to the first major branch point and performed volumetric analysis of territories supplied by individual third-order branches using Ziostation REVORAS. Hepatic vein anatomy was categorized as HV-type 1 (a fissure plane forming an intersegmental vein), HV-type 2 (a landmark vein without plane formation), or HV-type 3 (neither) and compared between portal types. **Results:** PV-type 1 was observed in 45 cases (45.0%), while PV-type 2 was seen in 53 cases (53.0%); however, two cases (2.0%) showed other patterns. The distance to the first branch point was shorter in PV-type 1 than in PV-type 2 (8.89 ± 7.87 vs. 17.3 ± 8.36 mm; *p* < 0.001). In PV-type 1, the first caudal branch territory (corresponding to S6) accounted for 43.8% ± 12.6% of the posterior section, whereas the first-branch cone-unit territory in PV-type 2 accounted for 14.9% ± 8.64% (*p* < 0.001). The HV-type 1/2/3 ratios differed by portal type (PV-type 1: 29/16/0; PV-type 2: 25/14/14; *p* < 0.001), and 26.4% of PV-type 2 cases lacked any hepatic venous landmarks. **Conclusions:** PV-type 2 peripheral RPPV branching was observed in 53% of cases. Although conventional Couinaud-based segmentectomy with the Glissonian approach and vein-guided transection was suitable for PV-type 1, cone-unit-based anatomical resection supported by individualized preoperative simulation and intraoperative navigation may prove useful for PV-type 2 anatomy.

## 1. Introduction

Couinaud’s description of functionally independent liver segments provides the conceptual foundation for anatomical liver resection and remains a standard framework in hepatobiliary surgery [1]. The Brisbane 2000 terminology harmonizes international nomenclature and reinforces the hierarchical view of liver division (first-order hemi-liver, second-order section, and third-order segment) [2,3]. However, this terminology did not define how segmental borders should be identified in vivo nor how anatomical units smaller than one segment should be described. The Tokyo 2020 update, which was developed by the Precision Anatomy for Minimally Invasive HBP Surgery (PAM-HBP) consensus, explicitly addressed anatomical resection for segment or less and introduced standardized terminologies for peripheral portal territories, such as the ‘cone unit’ [4].

Studies have also re-evaluated Couinaud’s segmentation, highlighting the need for a more detailed anatomical understanding. For example, some authors have proposed dividing the right anterior section into dorsal and ventral parts [5]. Furthermore, it has been shown that planes of the portal fissure often have plane-forming fissure vein branching across them, which can serve as guides for anatomical liver resections—an approach known as hepatic vein-guided anatomical liver resection [6,7,8].

Understanding the precise anatomy of the right posterior section is becoming increasingly important, as various surgical procedures involving this section, such as posterior sectionectomy, segment 6 or 7 segmentectomy, and posterior section grafts for living donor liver transplantation, have been developed and widely adopted, even as minimally invasive procedures [9,10]. Contrary to the traditional view, the right anterior and posterior sections are not simply divided into two equal parts by a vertical fissure plane, and significant anatomical variations in their segments have also been identified [7].

Although segment 7 segmentectomy is considered one of the most complex liver resections, especially in minimally invasive procedures, the precise anatomy of this section remains unclear and is certainly more complex than Couinaud’s simple definition of segments 6 and 7 (caudal and cranial parts of the posterior section) [7,8,9]. In some cases, the posterior portal branch divides directly into two major branches (segments 6 and 7). However, in other cases, multiple branches arise sequentially from a looped major posterior trunk, making it difficult to define a clear border between segments 6 and 7 [11]. As highlighted by Wakabayashi et al., Couinaud’s segmentation often fails to reflect the actual anatomical variations, particularly in the right posterior section. Their review emphasized that segmental borders are not fixed and require a combination of landmarks, such as hepatic veins, portal vein branching patterns, and intraoperative tools such as ultrasonography, indocyanine green (ICG) fluorescence, and three-dimensional (3D) simulations for accurate identification [4,12].

In the present study, we analyzed the branching patterns of the right posterior portal vein by jointly evaluating the hepatic vein, using computed tomography (CT)-based 3D reconstruction images, to establish a new sub-sectional anatomy of the posterior section. Thus, this study could be the basis for more precise anatomical liver resections of the right posterior section.

## 2. Materials and Methods

### 2.1. Materials

This study analyzed CT-based 3D reconstruction images, including those of portal and hepatic veins, in a consecutive series of 100 eligible cases at our institution (Fujita Health University Okazaki Medical Center).

Patient characteristics, including age, sex, BMI, disease, and surgical history, are summarized in Table 1.

All imaging data were obtained from five-phase contrast-enhanced CT scans performed at our institution for the preoperative planning of complex procedures of liver resection or pancreatectomy between April 2020 and October 2025. Among 200–250/year cases of cancer surgery that underwent preoperative evaluation with contrast-enhanced CT scans, 125 cases underwent detailed examination via five-phase contrast-enhanced CT scans during the period. Patients who underwent 5-phase contrast CT were selected based on their needs for preoperative simulations. Liver surgery patients who underwent complicated procedures, such as sectionectomy, segmentectomy, and deep small (less than segment) anatomical resection, were selected, as well as those with surgeries involving the pancreas or biliary tract who needed preoperative assessments given their extent of disease spreading.

Among those who underwent 5-phase contrast CT, patients with factors that can affect liver anatomy in the right posterior section were excluded from the analyses. Patients with giant tumors; liver tumors inside or next to the posterior section, which can distort the anatomy; and a prior liver resection history were excluded. No patients had severe liver cirrhosis and portal hypertension. Patients with conditions that can affect the anatomy of the Glissonian pedicle, such as portal vein thrombosis, biliary obstruction and dilatation, hilar tumors, and vascular invasion, were also excluded. Thus, a total of 25 patients were excluded.

### 2.2. Software and Reconstruction

Three-dimensional reconstruction and volumetric analyses were performed using Ziostation REVORAS (Version 5.2.1.0, Ziosoft, Tokyo, Japan), while portal and hepatic veins and liver parenchyma were extracted automatically using the software Ziostation REVORAS from 5-phase contrast CT images. Automatic segmentation was then performed based on the portal vein distribution determined by the software. Subsequently, the results were examined and confirmed independently by authors TI and ZM. All figures of portal and hepatic veins, the liver parenchyma, and its segmentations were manually corrected when needed after a consensus was reached between the two authors.

### 2.3. Analysis of Portal Vein Branching Patterns

**Figure 1:**
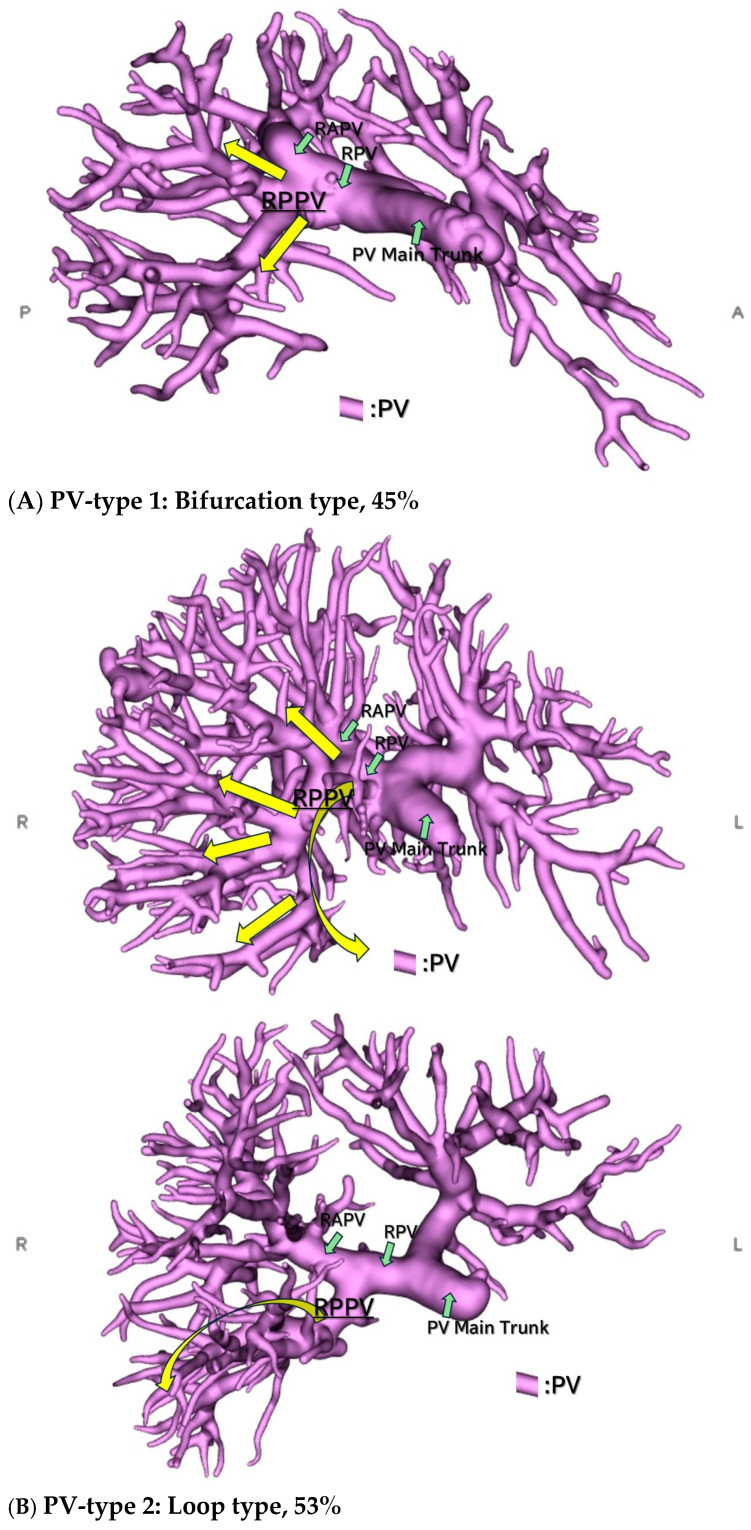

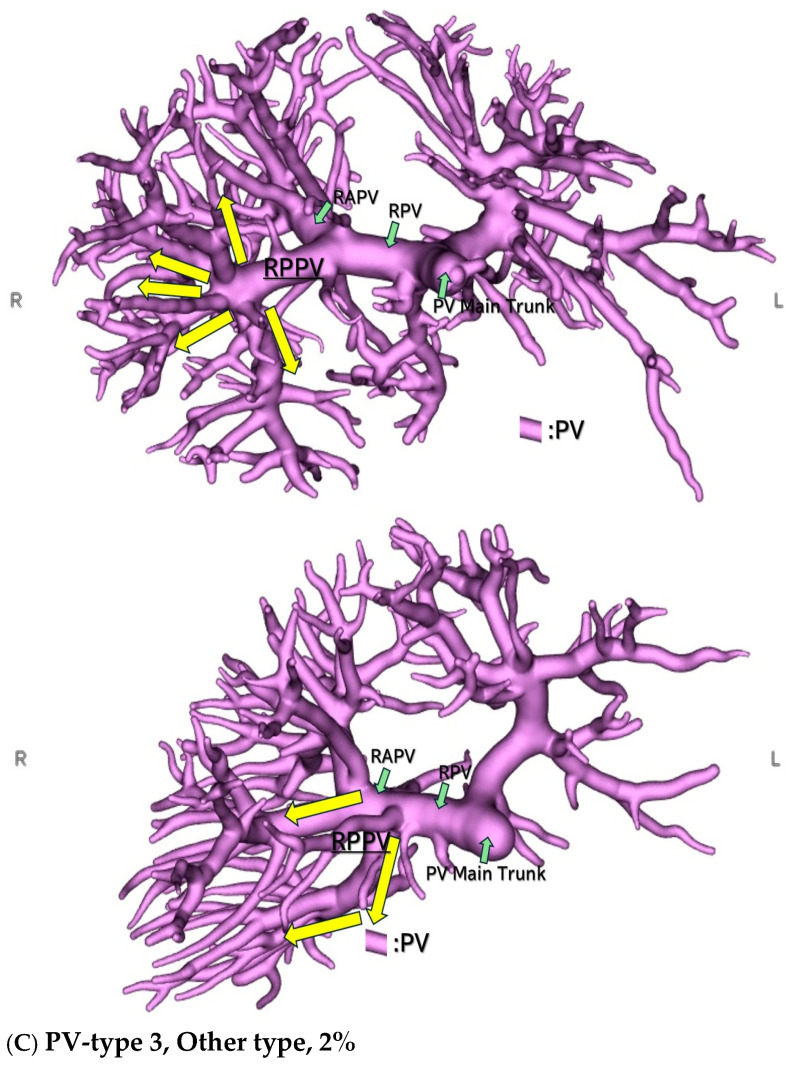
Types of portal vein branching in the right posterior section. PV main trunk: the main trunk of the portal vein; RPV: right portal vein; RAPV: right anterior portal vein; RPPV: right posterior portal vein; yellow arrows: flow of portal veins. (**A**) PV-type 1: Bifurcation type (*n* = 45, 45.0%), in which the RPPV divides into two major branches (G6 and G7) with subsequent peripheral arborization; the three-dimensional length of the common stem before the bifurcation is 8.89 ± 7.87 mm. (**B**) PV-type 2: Loop type (*n* = 53, 53.0%), in which the RPPV forms an arch-like loop issuing sequential small branches (cone units); the 3D distance from the RPPV origin to the first branch is 17.3 ± 8.36 mm, which is significantly longer than that in type 1. (*p* < 0.001). The convex loop mainly protruded to the right side (50/53, 94.3%) (upper), but protrusion on the left side (3/53, 5.7%) (lower) was occasionally observed. (**C**) PV-type 3: Other type (*n* = 2, 2.0%); in one case, the RPPV divides into multiple branches simultaneously after the common stem (upper); in the other case, one portal vein leading to segment 6 divides from the right anterior section of the portal vein (lower).

The branching patterns of the right posterior portal vein were examined using 3D reconstruction images, and third-order branches divided from the main posterior portal vein were analyzed to determine whether they could be classified into distinct types (PV-type 1 (bifurcation type), PV-type 2 (loop type), or PV-type 3 (others); Figure 1) [11]. Two examiners (TI and ZM) independently evaluated the reconstruction images and classified each case into one of three types. When there was a mismatch between them, the case type was defined after discussion with one additional examiner (KN).

When the first branch coursed directly toward the IVC, it was judged to be a caudate lobe branch and excluded. In those cases, the second branch was treated as the first posterior branch for computation. Additionally, only branches distributed along the dorsal–caudal area of the plane defined by the right hepatic vein and its branches were defined as branches in the posterior section.

### 2.4. Volumetric Measurement of Each Sub-Sectional Area

The volume of each sub-sectional area supplied by individual third portal branches in the posterior section was calculated using the volumetric function found in REVORAS and is expressed as follows:

- Milliliter of the area (mean ± SD);
- Ratio of the entire liver volume of the right posterior section (%, mean ± SD).

### 2.5. Measurement of 3D Distance from the Bifurcation to the First Branch

The 3D distance from the origin of the right posterior portal vein to its first branch was measured.

Distances were calculated by selecting three or more points on the vein on the CT scan and computing the spatial distance using the measurement tool in REVORAS.

### 2.6. Analysis of Hepatic Vein, as Intersegmental Fissure Vein (ISV) or Landmark of Segmentectomy

**Figure 2:**
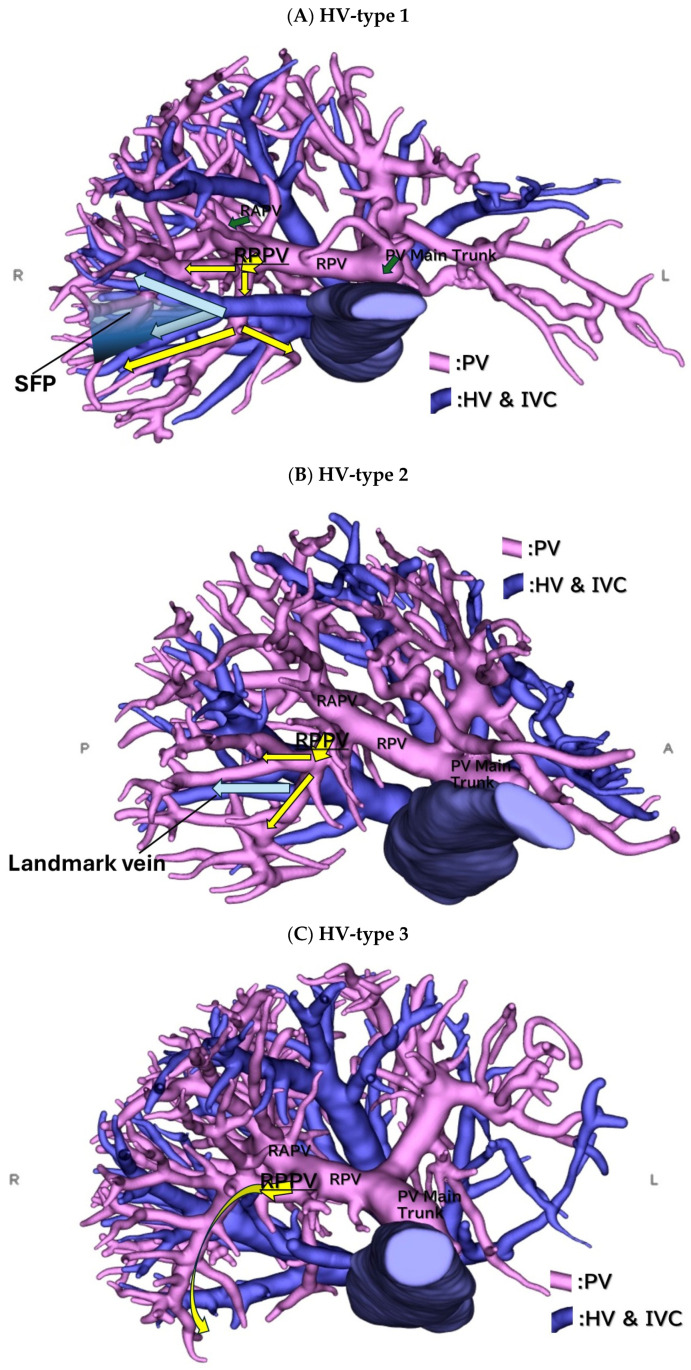
Types of hepatic vein branching in the right posterior section. PV main trunk, the main trunk of portal vein; RPV, right portal vein; RAPV, right anterior portal vein; RPPV, right posterior portal vein; yellow arrows, flow of portal veins; pale blue arrows, flow of hepatic veins; SFP, segmental fissure plane. (**A**) HV-type 1: The peripheral branches of the RHV or IRHV form a segmental fissure plane, branch over, and form a plane. (**B**) HV-type 2: The RHV, IRHV, or their branches can be a landmark for resections of segments 6 and 7 (caudal and cranial parts of the posterior section), but they do not form a segmental fissure plane. (**C**) HV-type 3: Veins are neither an ISV nor a landmark.

The branches of the right hepatic vein (RHV) or inferior right hepatic vein (IRHV) were analyzed. They were divided into three groups (Figure 2): HV-type 1, in which the peripheral branches of the RHV or IRHV form an intersegmental fissure plane between segments 6 and 7 (caudal and cranial parts of the posterior section); HV-type 2, in which the RHV, IRHV, or their branch can be a landmark for resections of segments 6 and 7 but do not form an intersegmental fissure plane; and HV-type 3, in which veins neither form an intersegmental fissure plane nor are a landmark of resection. The ratios of these hepatic vein types in each PV group were evaluated.

### 2.7. Statistical Analysis

Data are expressed as the mean ± standard deviation, median [IQR], or number (%) of patients. Continuous variables are summarized as the mean ± standard deviation if they are normally distributed (according to the Shapiro–Wilk test); otherwise, they are expressed as the median [IQR].

Between-group differences in categorical variables were analyzed using Pearson’s chi-squared test. Between-group differences in continuous parametric variables were analyzed using unpaired Student’s *t*-test, while between-group differences in continuous non-parametric variables were analyzed using the Mann–Whitney test. Statistical analyses were performed using SPSS Statistics version 28 (IBM Corp., Armonk, NY, USA), with significance set at *p* < 0.05.

### 2.8. Ethical Considerations

This study was approved by the Institutional Review Board of Fujita Health University (Approval No. HM24-029), and all procedures were conducted in accordance with the Declaration of Helsinki.

Given the retrospective nature of this study, no new data or specimens were collected, and only the existing data were analyzed. Therefore, information regarding the study was disclosed publicly, and participants were guaranteed the opportunity to refuse participation through an opt-out process. No informed consent was obtained from the participants.

This article is presented in accordance with the STROBE reporting checklist. The STROBE reporting checklist completed by authors is available in Supplementary Material.

## 3. Results

The branching patterns of the third-order portal branches in the right posterior section (RPPV) from 100 cases were categorized into three types [Figure 1].

For PV-type 1(Bifurcation type, *n* = 45, 45.0%), the RPPV divides into two major branches (G6 and G7: Glissonean pedicles to segments 6 and 7) with subsequent peripheral arborization; the three-dimensional length of the common stem before the bifurcation is 8.89 ± 7.87 mm.

For PV-type 2 (Loop type, *n* = 53, 53.0%), the RPPV forms an arch-like loop issuing sequential small branches (cone units), and the 3D distance from the RPPV origin to the first branch is 17.3 ± 8.36 mm, which is significant longer than that in PV-type 1 (*p* < 0.001) [Table 2].

PV-type 3 (Other type, *n* = 2, 2.0%): In one case, the RPPV is divided into multiple branches simultaneously after the common stem, and in the other case, one portal vein leading to segment 6 was divided from the right anterior section of the portal vein.

Based on volumetry calculated using Ziostation REVORAS, the volume of segment 6 (the area supplied by the first branch toward the right caudal direction) corresponds to 43.8% ± 12.6% of the whole right posterior section in PV-type 1 cases, while that of the first-branch cone-unit area corresponds to 14.9% ± 8.64% in PV-type 2. There was a statistically significant difference between the volumes (*p* < 0.001) [Table 2].

In cases with the loop type (PV-type 2), the convex loop mainly protrudes to the right side (50/53, 94.3%), with occasional protrusion to the left (3/53, 5.7%). In addition, there are many variations in the branching and distribution patterns of portal veins categorized as the third branch type. There are cases where caudal-to-cranial branches appear sequentially from the first branch and others where caudal and cranial branches appear randomly.

Analyses of the venous branching patterns showed that the ratio of HV-type 1:HV-type 2:HV-type 3 in each type of portal vein branching pattern was 29:16:0 (64.4%:35.6%:0%) in PV-type 1 and 25:14:14 (47.2%:26.4%:26.4%) in PV-type 2. In PV-type 1, the IRHV or the distal part of the RHV always lies between segments 6 and 7 as landmarks, and their tributaries more frequently delineate the intersegmental fissure plane between the caudal and cranial parts than those of PV-type 2 (*p* < 0.001). In PV-type 2, 26.4% of the patients do not have the vein as a landmark or a fissure vein [Table 3].

## 4. Discussion

In our cohort, the ratio (53%) of loop-type peripheral portal branching in the right posterior section observed reinforces prior imaging studies reporting that the classical Couinaud-style dichotomy into P6 and P7 branches is not the most common pattern [11]. Since the objective of this study was intended to lead to surgical planning implications, we additionally examined the other major factors resulting in more precise surgical implications by combining hepatic vein analysis, volumetric territory quantification, and distance measurements on previous reports. However, we only examined the portal vein, hepatic vein, and liver parenchyma as practical major factors and did not include the artery and bile duct in the first step. Minami et al. described an arch-like single-trunk loop supplying multiple branches in 50% of cases and a Couinaud-type bifurcation in 45% of CT-based reconstructions, closely mirroring our distribution [11]. Our data also show that when the first branch from the posterior trunk is divided earlier, the branching type tends to be the bifurcation type (*p* < 0.001). Furthermore, the volume of the area supplied by the first branch from the posterior trunk is much larger in PV-type 1 than in PV-type 2 (*p* < 0.001). These data also substantiate that there are two major branching patterns: (1) a short main posterior trunk that splits into two similar-level sub-trunks (caudal and cranial) and (2) a long looping main posterior trunk that divides into small branches sequentially.

Wang et al. identified an intersegmental fissure vein (ISV) between S6 and S7 in 82% of patients (derived from the RHV in 92% and the IRHV in 8%) and successfully implemented ISV-guided laparoscopic segmentectomies with favorable perioperative outcomes, including R0 rates [13]. However, in the present study, an ISV forming a fissure plane was not identified in 35.6% and 52.8% of cases in the bifurcation and loop types of portal vein branching patterns, respectively. Even when landmark veins (not forming the plane) were present, 26.4% of the loop-type cases did not have such veins. The present data indicate the need to reconsider sub-sectional anatomical resection in the posterior section. Although Couinaud’s classification of segments 6 and 7 can be applied to some parts of the patient, there are others with loop-type branching (some even without ISVs) who do not fall into it. Nevertheless, the strategy based on Couinaud’s classification (segments 6 and 7) using the Glissonian approach and/or a hepatic vein-guided approach is still valuable for almost half of the patients. However, others do not fit this strategy. In patients with loop-type PV branching without ISVs, “cone-unit resection” based on individualized preoperative planning and an intraoperative approach with navigation should be considered [12,14,15].

Anatomical resection depends on the Glissonian pedicle territory, which is not only observed in portal inflow and hepatic venous landmarks but also in arterial inflow and biliary drainage. However, this study, which aims to determine surgical implications, has only examined the portal vein, hepatic vein, and liver parenchyma as major factors, since the arterial distribution often has a complicated anatomy and CT-based cholangiography is not performed routinely in our institution to obtain detailed information on the biliary tract.

**Figure 3:**
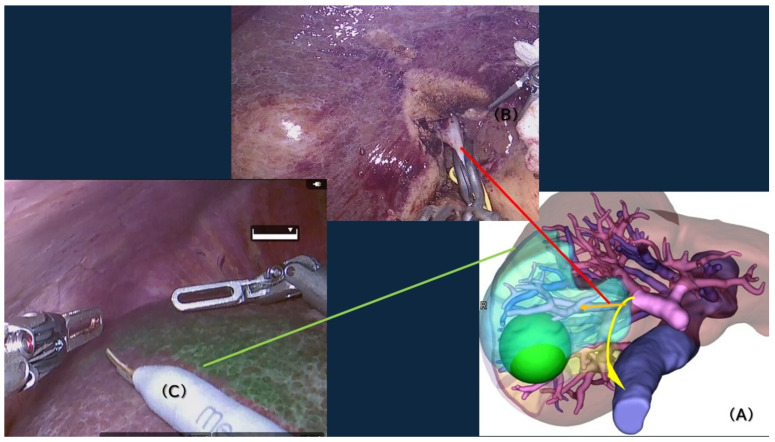
Simulation and navigation for liver resection. (**A**): The image of a preoperative CT simulation. Purple: hepatic veins and the vena cava; pink: portal vein; green: hepatocellular carcinoma; curved yellow arrow: looped posterior portal vein; ocher arrow: first 3rd-order branch from the posterior portal vein. The tumor was mainly located inside the first branch territory, and robotic resection of the extended territory was planned. (**B**) Intraoperative findings: The 1st-order branch from the posterior portal vein was dissected and clamped (red line). (**C**) ICG injection after branch clamping showed a clear demarcation line indicating the tumor-bearing area.

In the context of liver surgery in the posterior section, these findings suggest that separate approaches to sub-sectional anatomical liver resection may be appropriate for the right posterior section with bifurcation (PV-type 1) and loop (PV-type 2) types. In cases with the bifurcation type (PV-type 1), conventional Couinaud-based segmentectomies (S6 and S7) augmented by the Glissonian approach and/or ISV-guided parenchymal transection remain appropriate. However, in loop-type (PV-type 2) anatomy, cone-unit-based anatomical resection, defined by peripheral portal territories, may prove useful. In those cases, translating preoperative territory maps into intraoperative planes during surgery planned with the cone-unit strategy requires complementary modalities. When the root of the target Glissonian pedicle is deep and peripheral, and of the loop type (PV-type 2), access to the target Glissonian pedicle can only be obtained after parenchymal transection. The appropriate transection (portal fissure) plane should be set by searching for the distribution of portal veins under intraoperative ultrasonography while referencing liver surface landmarks and preoperative simulation images; however, ICG injection into the territorial portal vein can be occasionally used when possible. Reviews have summarized how indocyanine green (ICG) fluorescence can demarcate hepatic segments either by portal injection (positive staining) or intravenous injection after inflow control (negative staining) [16,17,18] (Figure 3). Prospective evidence suggests that ICG-guided recognition of boundaries is feasible and may reduce major liver-related complications in selected cohorts undergoing anatomical resection [19]. Additionally, real-time virtual sonography and fusion navigation systems can co-register intraoperative ultrasound with preoperative CT/3D simulations to navigate the planned planes in real time [20].

**Figure 4:**
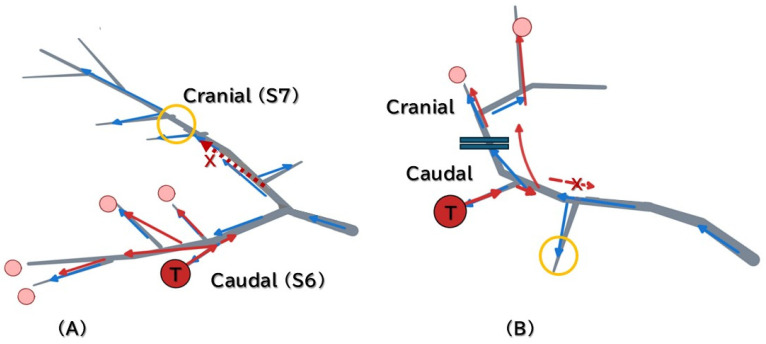
Theoretical schema of the difference in tumor spreading between those of the bifurcation and loop types. (**A**): Bifurcation type and (**B**): Loop type. The red colored-in circle with “T” shows a tumor (liver cancer). The pink colored-in circle shows possible tumor dissemination. The blue arrow shows the original flow of the portal vein. The red arrow shows possible drainage flow from the tumor-bearing area. The red interrupted arrow with “X” shows that a tumor cell does not spread in that direction. The yellow circle shows the area spared from tumor cell spreading. In bifurcation-type cases, tumor spreading first occurs inside the main third branch area (segment 6: caudal or segment 7: cranial). In loop-type cases, the most central branch of the portal vein is spared from tumor cell spreading, even though the branch and the tumor-bearing branch are both in the caudal part of the posterior section next to each other.

The concept of anatomical resection of the liver originates from the fact that the portal vein sometimes works as a drainage vein when huge HCC tumors compress the hepatic vein and disturb blood drainage through it. In this situation, tumor cell dissemination can occur in the portal territory [21,22]. Thus, surgical strategies for liver resection in bifurcation (PV-type 1) and loop (PV-type 2) types can be different. The cranial area of bifurcation-type (S7) veins in the posterior section is a separate independent portal territory in which the tumor cells can be disseminated. However, theoretically, the cone units of the loop type (PV-type 2) are separately and directly drained into the posterior main trunk, and tumor cells in one cone unit cannot be drained into the next unit, even if they are both in the same (cranial or caudal) half of the posterior section (Figure 4).

ISV-guided parenchymal transection is useful in most bifurcation-type cases. However, ISV branches are not always distributed on the fissure plane. There are cases in which a vein exists on the border between segments 6 and 7 and can be used as a landmark for resection; however, it does not form the fissure plane. In addition, the ISV is sometimes a peripheral branch of the RHV, but in other cases, it is that of the IRHV. The convex of the loop does not always protrude to the right side, with protrusion sometimes occurring to the left. There are various branching and distribution patterns of the portal and hepatic veins inside the posterior section, and variance in the right posterior venous outflow (RHV/IRHV complex) influences technical planning, particularly for S7 resections and posterior-section living donor grafts. Additionally, 3D donor analyses showed an inverse correlation between RHV and IRHV calibers and have led to the classification of multiple posterior venous configurations. IRHV incidence also negatively correlates with RHV diameter in CT-based reconstruction studies, offering predictive utility for venous preservation and reconstruction [23,24,25]. Individual plans, which are set by preoperative simulations based on imaging studies, and their implications with intraoperative navigation are needed.

The strengths of this study include “case-wise 3D interrogation of third-order and more distal portal branches with quantitative volumetry and distance measurement” and “pattern analyses of branching and distribution with the combination of portal and hepatic veins.” These directly translate and lead to an operative strategy.

Limitations include the single-center design and exploratory nature of venous mapping. This study’s cohort does not describe a healthy cohort; instead, it refers to patients requiring detailed preoperative assessment. Although the subjects were cautiously selected, their conditions, such as tumors and chronic liver disease, could influence the results. Although the subjects in this study are solely Japanese, our findings on anatomical proportions are expected to be universal even in Western populations given the data of previous portal vein studies [26]. Moreover, comprehensive integration of arterial and biliary variants relevant to full anatomical planning is not performed in this study. Anatomical resection depends not only on portal and hepatic veins but also on arterial inflow and biliary drainage. However, this study, which aims to determine surgical implications as its first step, has only examined portal and hepatic veins as major factors. Thus, these limitations should be addressed in future studies.

## 5. Conclusions

CT 3D reconstruction of the peripheral right posterior portal system demonstrated that a clear-cut dichotomy into S6 and S7 (PV-type 1) was present in less than half of the cases and that 53% of the cases present with loop-type (PV-type 2) ramification. Couinaud-based anatomical resections were most suitable for bifurcation-type (PV-type 1) cases. On the other hand, loop-type (PV-type 2) anatomy is often described without intersegmental veins from the RHV/IRHV system. For those cases, “cone unit” anatomical resections using preoperative simulation and intraoperative navigation may prove useful.

## Figures and Tables

**Table 1:** Patient characteristics. Values are expressed as a median [IQR] or number (%).

Characteristics	Total (*n* = 100)
Age (years), median [IQR]	71.0 [64.0–77.2]
Sex (male/female), *n*/*n* (%)	65/35 (65%/35%)
BMI (kg/m^2^), median [IQR]	22.3 [20.3–24.0]
Primary disease, *n* (%)	
Pancreatic cancer	22 (22.0%)
Biliary tract cancer	20 (20.0%)
Colorectal liver metastasis	19 (19.0%)
Hepatocellular carcinoma	13 (13.0%)
Others	26 (26.0%)
Previous abdominal surgery (yes/no), *n*/*n* (%)	28/72 (28%/72%)

**Table 2:** Quantitative characteristics of right posterior portal branching patterns (PV-type 1 vs. PV-type 2). Distance definitions: In PV-type 1, the “first branch point” refers to bifurcation into the major G6 and G7 pedicles; in PV-type 2, it refers to the first cone-unit branch. RPPV: right posterior portal vein. Values are expressed as the mean ± SD or number (%).

	PV-Type 1: Bifurcation (*n* = 45)	PV-Type 2: Loop (*n* = 53)	*p* Value
Distance from the RPPV origin to the first branch point (mm)	8.89 ± 7.87	17.3 ± 8.36	<0.001
Proportion of the first-branch territory within the right posterior section (%)	43.8 ± 12.6 (Segment 6 territory)	14.9 ± 8.64 (1st cone-unit territory)	<0.001
Loop convex direction	—	Right: 50 (94.3%); Left: 3 (5.7%)	—

**Table 3:** Distribution of hepatic vein patterns by portal branching type. Values are expressed as a number (%). * Pearson’s chi-squared test across the HV-type distribution.

Portal Branching Type	HV-Type 1 (Fissure-Plane-Forming), *n* (%)	HV-Type 2 (Landmark Only), *n* (%)	HV-Type 3 (Neither), *n* (%)	*p* Value *
PV-type 1: Bifurcation (*n* = 45)	29 (64.4%)	16 (35.6%)	0 (0%)	<0.001
PV-type 2: Loop (*n* = 53)	25 (47.2%)	14 (26.4%)	14 (26.4%)	

## Data Availability

The dataset is available upon request from the authors, and the raw data supporting the conclusions of this article will be made available by the authors upon request.

## Supplementary Materials

The following supporting information can be downloaded at: https://www.mdpi.com/article/10.3390/jcm15156093/s1. The authors completed the STROBE reporting checklist, which is available in Supplementary Material. This article is presented in accordance with the STROBE reporting checklist.
